# Late-onset Complications in a Chronic Proton Pump Inhibitor User: Electrolyte Abnormalities and a Diagnostic Approach

**DOI:** 10.1210/jcemcr/luaf125

**Published:** 2025-06-09

**Authors:** Danielle H Tran, Jason R Fredrick, Radhika R Narla

**Affiliations:** Department of Medicine, University of Washington, Seattle, WA 98195-6421, USA; Division of Metabolism, Endocrinology and Nutrition, Department of Medicine, University of Washington Medical Center, Seattle, WA 98195-6421, USA; Division of Metabolism, Endocrinology and Nutrition, Department of Medicine, University of Washington Medical Center, Seattle, WA 98195-6421, USA

**Keywords:** hypocalcemia, hypomagnesemia, hypokalemia, proton pump inhibitor, electrolyte disturbances

## Abstract

This case presents an unusual presentation of severe hypocalcemia, hypomagnesemia, and hypokalemia that suddenly developed in a patient with a prior 15-year history of proton pump inhibitor (PPI) use with no prior complications. The patient developed acute onset of persistent tingling and numbness in her fingertips followed by acute confusion and perioral tingling. Upon hospitalization, she was found to have severe hypocalcemia, hypomagnesemia, and hypokalemia and required IV electrolyte repletion. This episode recurred, prompting the discontinuation of her PPI therapy. This unusual timing prompted an extensive workup to exclude alternative etiologies of hypocalcemia. Ultimately, PPI-induced hypomagnesemia was identified as the primary driver of the patient's electrolyte abnormalities. This case serves dual teaching points. First, it underscores the importance of recognizing that refractory hypocalcemia and hypokalemia can often be linked to untreated hypomagnesemia so that timely diagnosis, effective management, and minimization of hospitalizations can be ensured. Second, this case highlights the unusual timing of symptomatic hypomagnesemia and subsequent hypocalcemia and hypokalemia despite years of prior PPI usage without prior known complications.

## Introduction

Proton pump inhibitors (PPIs) are widely used for managing acid-related conditions such as gastroesophageal reflux disease (GERD), peptic ulcer disease, and functional dyspepsia [1-3]. Despite their widespread use and effectiveness, PPIs can lead to adverse effects, including hypomagnesemia, first reported in 2006 in 2 patients [4-9]. Subsequent studies, including a meta-analysis, have supported this association, noting a higher prevalence of hypomagnesemia in PPI users (19.4%) compared to nonusers (13.5%), with an adjusted odds ratio of 1.71 (95% confidence interval 1.33, 2.19) [10-15]. However, the risk remains debated due to heterogeneity among studies.

The suspected mechanism involves impaired magnesium absorption rather than increased renal loss. PPIs may inhibit magnesium transport through active inhibition of transient receptor potential melastatin (TRPM6 and TRPM7) channels in the duodenum and passive inhibition of paracellular pathways in the intestine [16, 17]. PPI-induced hypomagnesemia can lead to secondary electrolyte disturbances, including hypokalemia, due to potassium efflux via renal medullary outer potassium channels, and hypocalcemia via mechanisms such as reduced calcium bioavailability from suppressed gastric acid production, impaired PTH secretion and resistance, and altered vitamin D metabolism [18, 19]. Treatment involves magnesium repletion, correction of associated electrolyte disturbances, and discontinuation of PPIs.

We report an unusual case of a patient with a 15-year history of PPI use who experienced sudden, severe hypomagnesemia, hypocalcemia, and hypokalemia on 2 occasions.

## Case Presentation

The patient is a 43-year-old female with a history significant for hyperlipidemia, hypertension, inflammatory bowel syndrome, hiatal hernia, and GERD on PPI since 2008. She developed acute confusion, perioral tingling, and weakness in June 2023, requiring presentation to the emergency department (ED) and her first inpatient admission. At that time, she had no prior history of inpatient admissions for any medical conditions, and no relevant family history was noted including no parathyroid problems.

## Diagnostic Assessment

**Figure 1.**
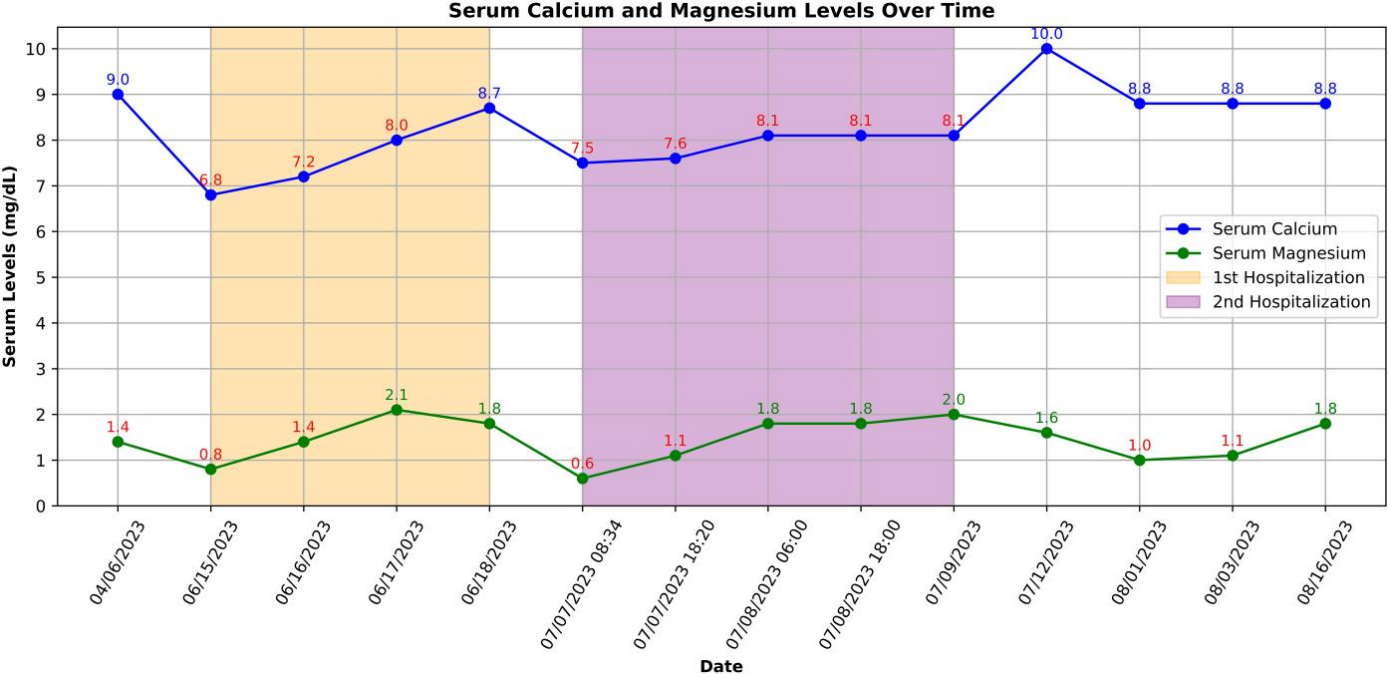
Scatter plot showing the relationship between serum calcium and magnesium levels with abnormal values in red.

Upon chart review and history taking with the patient, her onset of symptoms started a few months back in April 2023 before she presented in June 2023. During a routine primary care physician (PCP) visit in April 2023, she had reported nonspecific muscle weakness, cramps, and numbness in her limbs to her PCP. Initial outpatient laboratory testing with PCP revealed magnesium 1.4 mg/dL (0.58 mmol/L) (reference range: 1.6-2.5 mg/dL; 0.66-1.03 mmol/L) and potassium 3.0 mEq/L or mmol/L (reference range: 3.5-5.3 mEq/L or mmol/L). Other tested labs were within normal limits, including serum calcium 9.0 mg/dL (2.25 mmol/L) (reference range: 8.4-10.2 mg/dL; 2.10-2.25 mmol/L), albumin 4.4 g/dL (44 g/L) (reference range: 3.5-5.2 g/dL; 35-52 g/L), and vitamin D 1,25 OH level 30.1 pg/mL (72.24 pmol/L) (reference range: 24.8-81.5 pg/mL; 59.52-195.60 pmol/L) (Table 1, Fig. 1). She was placed on oral supplementation of unclear dosages and without repeat laboratory follow up. She presented to the ED in June 2023 with symptoms of neuromuscular instability. Physical exam noted on ED notes was negative for Chvostek's sign. Her labs revealed low levels of calcium 6.8 mg/dL (1.70 mmol/L) (reference range: 8.7-10.4 mg/dL; 2.17-2.6 mmol/L), magnesium 0.8 mg/dL (0.33 mmol/L) (reference range: 1.6-2.6 mg/dL; 0.66-1.07 mmol/L), and potassium 2.4 mEq/L or mmol/L (reference range: 3.5-5.1mEq/L or mmol/L), with normal levels of PTH 54 pg/mL or ng/L (reference range: 19-88 pg/mL or ng/L), vitamin D 25-OH 35 pg/mL (84 pmol/L) (reference range: 30-130 pg/mL; 72-312 pmol/L), and albumin 4.2 g/dL (42 g/L) (reference range: 3.2-4.8 g/dL; 32-48 g/L). She was admitted for IV electrolyte repletion. After IV repletion, she had resolution of her symptoms. No further workup was initiated other than asking her to consider holding her omeprazole. She was discharged with oral electrolyte supplementation without subsequent lab monitoring.

**Table 1.** **Laboratory results for our patient with chronic proton pump inhibitor usage**.

Hospitalization	Date	Serum calcium	Serum albumin	Serum magnesium	Serum potassium	Serum PTH	Serum vitamin D 25-hydroxy
		Reference ranges					
		8.4-10.2 mg/dL (2.10-2.55 mmol/L)	3.5-5.2 g/dL (35.00-52.00 g/L)	1.6-2.5 mg/dL (0.66-1.03 mmol/L)	3.5-5.1 mEq/L (3.50-5.10 mmol/L)	15-65 pg/mL (15-65 ng/L)	30-100 ng/mL (74.88-249.60 nmol/L)
	04/06/2023	9.0 mg/dL (2.25 mmol/L)	4.4 g/dL (44.00 g/L)	**1.4 mg/dL (0.58 mmol/L)**	**3.1 mEq/L (3.10 mmol/L)**	NA	30.1 ng/mL (75.13 nmol/L)
First hospitalization	06/15/2023	**6.8 mg/dL (1.70 mmol/L)**	4.5 g/dL (45 g/L)	**0.8 mg/dL (0.33 mmol/L)**	**2.4 mEq/L (2.40 mmol/L)**	54 pg/mL (54 ng/L)	35 ng/mL (87.50 nmol/L)
	06/16/2023	**7.2 mg/dL (1.79 mmol/L)**	4.2 g/dL (42 g/L)	**1.4 mg/dL (0.58 mmol/L)**	**2.8 mEq/L (2.80 mmol/L)**	NA	NA
	06/17/2023	**8.0 mg/dL (2.0 mmol/L)**	NA	2.1 mg/dL (0.86 mmol/L)	**3.3 mEq/L (3.30 mmol/L)**	NA	NA
	06/18/2023	8.7 mg/dL (2.17 mmol/L)	NA	1.8 mg/dL (0.74 mmol/L)	4.4 mEq/L (4.40 mmol/L)	NA	NA
Second hospitalization	07/07/2023 08:34	**7.5 mg/dL (1.88 mmol/L)**	4.1 g/dL (41.00 g/L)	**0.6 mg/dL (0.25 mmol/L)**	**2.6 mEq/L (2.60 mmol/L)**	31 pg/mL (31.00 ng/L)	47.1 ng/mL (117.56 nmol/L)
	07/07/2023 18:20	**7.6 mg/dL (1.90 mmol/L)**	3.8 g/dL (38.00 g/L)	**1.1 mg/dL (0.45 mmol/L)**	NA	NA	NA
	07/08/2023 06:00	**8.1 mg/dL (2.02 mmol/L)**	NA	1.8 mg/dL (0.74 mmol/L)	NA	NA	NA
	07/08/2023 18:00	**8.1 mg/dL (2.02 mmol/L)**	NA	1.8 mg/dL (0.74 mmol/L)	NA	NA	NA
	07/09/2023	**8.1 mg/dL (2.02 mmol/L)**	NA	2.0 mg/dL (0.82 mmol/L)	NA	NA	NA
	07/12/2023	10.0 mg/dL (2.50 mmol/L)	NA	1.6 mg/dL (0.66 mmol/L)	4.4 mEq/L (4.40 mmol/L)	NA	NA
	08/01/2023	8.8 mg/dL (2.20 mmol/L)	4.4 g/dL (44.00 g/L)	**1.0 mg/dL (0.41 mmol/L)**	**2.8 mEq/L (2.80 mmol/L)**	61 pg/mL (61.00 ng/L)	50.1 ng/mL (125.05 nmol/L)
	08/03/2023	8.8 mg/dL (2.20 mmol/L)	NA	**1.1 mg/dL (0.45 mmol/L)**	NA	37 pg/mL (37.00 ng/L)	44.2 ng/mL (110.32 nmol/L)
	08/16/2023	8.8 g/dL (2.20 mmol/L)	NA	1.8 mg/dL (0.74 mmol/L)	**3.2 mEq/L (3.20 mmol/L)**	NA	NA

Abnormal values in bold.

Abbreviation: NA, not available.

Several weeks later, in July 2023, in addition to running out of her oral supplementation, she also resumed taking omeprazole from 40 mg daily to twice daily, again due to unrelenting GERD symptoms. A few days after restarting her PPI, she experienced reoccurring numbness and tingling and was readmitted a second time for severe electrolyte deficiencies. At the time of the second hospitalization, her serum calcium was 7.5 mg/dL (1.88 mmol/L), magnesium 0.6 mg/dL (0.25 mmol/L), and potassium 2.6 mEq/L or mmol/L. Albumin was 4.1 g/dL (41 g/L), and ionized calcium was 1.9 mEq/L (0.47 mmol/L) (reference range: 2.24-2.60 mEq/L; 0.56-0.65 mmol/L). PTH was 31 pg/mL or ng/L (reference range: 15-65), and vitamin D 25-OH level was 44.6 ng/mL (111.32 nmol/L) (reference range: 30-100; 74.88-249.6 nmol/L) (Table 1, Fig. 1). Serum phosphorus during this admission ranged from low to normal, measuring at 3.7 mg/dL (1.2 mmol/L) (reference range: 2.7-4.5; 0.87-1.45 mmol/L) on admission and 2.0 mg/dL (0.65 mmol/L) upon discharge. She was treated with IV repletion of her electrolytes during her second hospitalization. Due to clinical suspicion that her PPI was the cause of her electrolyte abnormalities, she was told to discontinue her PPI and was started on maximum-dose famotidine therapy for her GERD. In addition, daily oral electrolyte supplementation was continued upon discharge.

**Figure 2.**
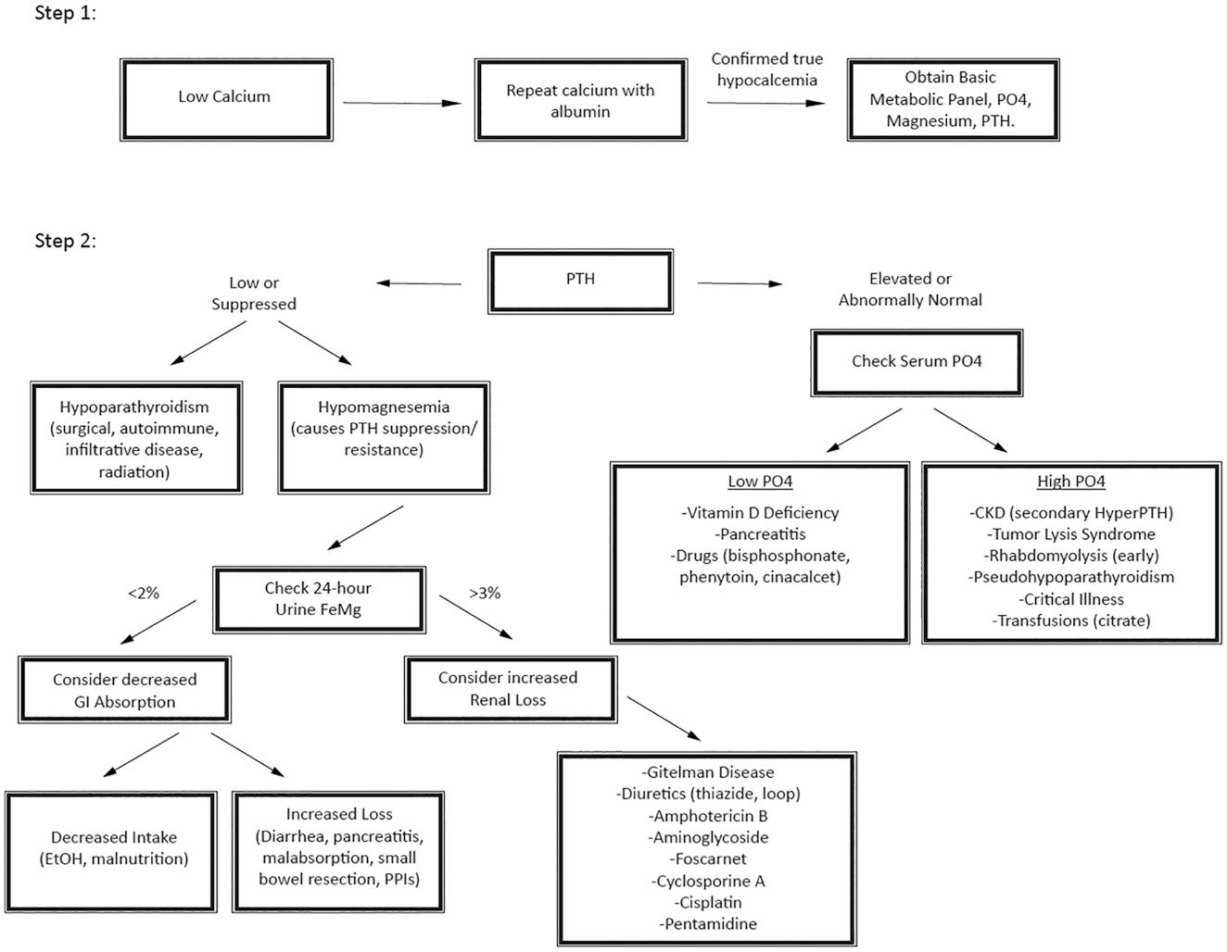
The diagnostic approach to hypocalcemia can begin with confirming calcium levels, correcting for albumin, and measuring PTH, phosphate, and serum magnesium. If magnesium is normal, further steps should be guided by the PTH value. However, if magnesium is low, repletion should be prioritized, followed by monitoring for calcium improvement. If hypocalcemia resolves with magnesium repletion, a 24-hour urine magnesium collection can help distinguish between renal wasting and reduced gastrointestinal absorption.

After her second hospitalization, she was referred to the endocrinology clinic with the posed clinical question focused on the workup of recurrent hypocalcemia (Fig. 2). From her history, our patient does not have a prior surgical history, such as thyroidectomy, parathyroidectomy, or radiation to the neck. She does not have a family history of genetic disorders. Therefore, in the context of her history and initial PTH being inappropriately normal despite concomitant hypocalcemia led to suspicion for non-PTH mediated causes [20]. Upon review of the patient's prior medical history, she had been on omeprazole 40 mg daily since 2008 for her chronic GERD but transitioned to twice daily dosing a year before her first admission, when her GERD symptoms were progressing. She did not have any other significant medication history, including no history of using bone resorption inhibitors (eg, bisphosphonates, calcitonin, denosumab) or gadolinium-based contrast agents (eg, gadodiamide, gadoversetamide) that may cause spurious hypocalcemia by interfering with the laboratory measurement of total calcium, calcium chelators (eg, EDTA, citrate, phosphate), diuretics, antibiotics (eg, aminoglycosides), or antifungals (eg, amphotericin, pentamidines).

The hypomagnesemia gave a clue to the leading cause of the refractory hypocalcemia, with PTH impaired secretion and resistance from hypomagnesemia (Fig. 2). Upon investigating the causes of hypomagnesemia, we found that although she does have irritable bowel syndrome, she does not have a history of chronic diarrhea, steatorrhea, small bowel bypass surgery, or severe alcohol use. In addition, iron studies, folate level, and vitamin B12 levels were also checked and normal, suggesting against general malabsorption. She had hypokalemia on presentation as well, further fueling the clinical suspicion that the cause may be related to PPI. However, given the unique timing of these electrolyte abnormalities in a patient who had been on a PPI without prior complications for 15 years, nephrology was consulted regarding the differential of a renal wasting pathology. Her 24-hour urine studies showed appropriately low urine calcium <1.2 mg/L (<0.30 mmol/L) and urine magnesium <1.8 mg/dL (<0.74 mmol/L), suggesting appropriate renal handling. They concurred that the PPI usage was the presumed etiology of hypomagnesemia, also contributing to her hypocalcemia and hypokalemia. The PTH levels were inappropriately normal with hypocalcemia, also reported as a rare occurrence by others in case reports, likely due to impaired PTH secretion and resistance from hypomagnesemia [21].

## Treatment

In April 2023, she was reportedly started on oral electrolyte supplementation of unknown dosing. In June 2023, during her first admission at an outside hospital, she had IV repletion of her electrolytes. She was discharged on oral calcium carbonate 500 mg 3 times daily, magnesium oxide 420 mg daily, and potassium chloride 20 mEq twice daily, but she ran out of her supplements 5 days before presenting to her second hospitalization. During her second admission, she was repleted with IV calcium carbonate 0.7 gm/D5W, oral potassium chloride 40 mEq, and IV magnesium sulfate 4 mg in the ED. Her home oral supplements were then continued for the rest of the second admission. She was discharged on the same previous dosing of her home potassium, magnesium, and calcium supplements, but the key treatment decision was to counsel her to formally discontinue her PPI.

## Outcome and Follow-up

Several days after her second discharge on July 12, 2023, her labs remained stable with serum calcium 10.0 mg/dL (2.5 mmol/L), magnesium 1.6 mg/dL (0.66 mmol/L), and potassium 4.4 mEq/L or mmol/L. She remained on daily oral electrolyte supplementation and off her PPI. On her last follow-up on August 16, 2023, her recheck labs showed improvement of magnesium 1.8 mg/dL (0.74 mmol/L), potassium 3.2 mEq/L or mmol/L, and stable calcium 8.8 mg/dL (2.2 mmol/L).

She reported that after being started on oral supplementation and discontinuing her PPI, she has not reexperienced numbness or tingling in her limbs.

## Discussion

Our patient developed sudden electrolyte abnormalities despite stable levels during 15 years of PPI usage. This resulted in 2 hospitalizations before endocrinology was consulted to rule out alternative causes of hypocalcemia. After workup, it was concluded that the recurrent hypocalcemia as well as hypokalemia was due to hypomagnesemia from PPI usage.

Hypomagnesemia is a known complication of PPI usage [9-15]. However, it is notable that our patient developed sudden symptomatic hypomagnesemia and subsequent hypocalcemia and hypokalemia despite 15 years of prior PPI usage without known complications. A systematic review by Hess et al reported a median onset of PPI-induced hypomagnesemia of 5.5 years, with a range of 14 days to 13 years [22]. Another case series reported no electrolyte abnormalities observed with short-term PPI treatment, and the earliest observed PPI-induced hypomagnesemia was after 1 year of PPI use [23]. Prolonged PPI use may lead to gradual depletion of total body magnesium without detectable changes in ionized magnesium levels [24]. The increase in omeprazole dosage about a year prior for our patient may have been the precipitant to the electrolyte abnormalities; however, without consistent lab data preceding her presentation, a trend cannot be confirmed.

Lastly, given the unique timing and chronicity of PPI use in this patient's presentation, the endocrine team relied on a pragmatic and systematic approach to evaluating hypocalcemia. The diagnostic approach to hypocalcemia should begin with confirming calcium levels; correcting for albumin; and measuring PTH, phosphate, and serum magnesium (Fig. 2) [20]. If magnesium is normal, further steps should be guided by the PTH value. However, if magnesium is low, repletion should be prioritized, followed by monitoring for calcium improvement. If hypocalcemia resolves with magnesium repletion, a 24-hour urine magnesium collection can help distinguish between renal wasting and reduced gastrointestinal absorption. Understanding that magnesium depletion impairs PTH synthesis, secretion, and action—resulting in inappropriately low or “normal” PTH levels—is essential for timely diagnosis, effective treatment, and minimization of hospitalizations.

## Learning Points

- Electrolyte abnormalities have been documented to occur at any time from the onset of PPI usage, from 14 days to our patient who used a PPI for 15 years before a complication.
- Understanding and recognizing the diagnosis, workup, pathophysiology, and management of hypocalcemia, including evaluating for hypomagnesemia, are critical for ensuring patient safety and effective treatment plans.
- Magnesium depletion can cause impaired synthesis, secretion, and end-organ resistance to PTH. It is important to be aware that magnesium remains a separate lab order in most laboratories.

## Data Availability

Original data generated and analyzed for this case report are included in this published article.
